# Seroprevalence survey of Hepatitis E Virus in Domestic Pigs in Guangdong, China

**DOI:** 10.3390/ani14131861

**Published:** 2024-06-24

**Authors:** Dingyu Liu, Baoling Liu, Zhenwen He, Changhong Qiao, Qin Luo, Xiangyu Chen, Xiaohu Wang, Hua Xiang, Jing Chen, Pian Zhang, Yuan Huang, Gang Wang, Chen Tan, Rujian Cai

**Affiliations:** 1 Key Laboratory of Livestock Disease Prevention of Guangdong Province, Key Laboratory for Prevention and Control of Avian Influenza and Other Major Poultry Diseases, Ministry of Agriculture and Rural Affairs, Institute of Animal Health, Guangdong Academy of Agricultural Sciences, Guangzhou 510640, China; ytdl@webmail.hzau.edu.cn (D.L.);; 2 College of Veterinary Medicine, Huazhong Agricultural University, Wuhan 430070, China; 3 College of Animal Science and Technology, Zhongkai University of Agriculture and Engineering, Guangzhou 510225, China

**Keywords:** HEV, zoonoses, pigs, seroprevalence, ELISA

## Abstract

**Simple Summary:**

As a zoonotic pathogen, the Hepatitis E virus (HEV) is extensively distributed worldwide. Animal infections are typically asymptomatic, while human infections can result in both acute and chronic viral hepatitis. The transmission pathway is typically a fecal–oral one. Pigs are the primary host of HEV genotype 4 (HEV-4), which primarily causes sporadic infections in China. The aim of this study was to evaluate HEV infection in certain farms in Guangdong, China. Twenty-five pig farms provided 1568 blood samples, of which 902 (902/1568, 57.53%) were positive for anti-HEV IgG. Such a high serum positive rate indicates that pigs in Guangdong are widely exposed to HEV, which is a considerable public health and safety concern.

**Abstract:**

The Hepatitis E virus (HEV) causes acute and chronic Hepatitis E and is a global public health concern. HEV genotypes 3 (HEV-3) and 4 (HEV-4) are common to humans and animals, and domestic pigs and wild boars have been identified as the main reservoirs. However, limited information is available on the status of HEV infection in pigs, particularly in the Guangdong Province, China. This study aimed to investigate the seroprevalence of HEV in pig farms within the Guangdong Province. A total of 1568 serum samples were collected from 25 farms and tested for anti-HEV IgG antibodies. Enzyme-linked immunosorbent assay (ELISA) results revealed that 57.53% (902/1568) of serum samples from 24 farms (24/25, 96%) were positive for anti-HEV IgG antibodies. Year, season, region, and age were all linked risk factors for HEV in Guangdong, with season and region showing more significant impacts. The results showing a high seroprevalence of HEV confirmed its circulation among domestic pigs in the Guangdong Province, China. The presence of this antibody indicates that HEV infection was or is present on farms, posing a risk of zoonotic transmission of HEV from pigs to exposed workers and from pork or organs to consumption.

## 1. Introduction

The Hepatitis E virus (HEV) is a leading cause of acute hepatitis worldwide. Hepatitis E infection in healthy humans is usually self-limiting. However, individuals with immunocompromised systems, co-infections, or organ transplants may develop fulminant infections and are at a high risk of developing chronic diseases. The case fatality rate of pregnant women infected with HEV is as high as 30%, compared with 1–2% in the general population [1,2].

HEV belongs to a family of *Hepatoviruses*, which are small icosahedral viruses categorized into *Orthohepevirus* and *Parahepevirinae*. *Orthohepevirus* is classified into four genera, among which *Paslahepevirus* infects humans and mammals [3]. HEVs under *Paslahepevirus* can be categorized into eight genotypes, namely, HEV-1 to HEV-8, and these genotypes are linked to various hosts and are distributed worldwide. HEV-1 and HEV-2 are found only in humans and are transmitted through water sources [4]. HEV-3 is currently the most common HEV genotype in the world; it is widely distributed in both industrialized and developing nations. It is present in humans and other species, and foodborne infections are its primary means of transmission in humans [5]. HEV-4 is mainly found in Asia, particularly in China, Japan, Vietnam, and Thailand. Similar to HEV-3, HEV-4 is a zoonotic pathogen that mostly infects people via foodborne means [6]; HEV-5 and HEV-6 are mainly associated with wild boars in Africa [7]; and HEV-7 and HEV-8 are mainly associated with camels [8]. HEV-induced hepatitis is a serious public health concern [9]. Data published by the Chinese Center for Disease Control and Prevention show that HEV cases accounted for 1 in 1000 of all infectious diseases between 2010 and 2020 [10]. A survey that included 208 studies published between 1997 and 2022 in Chinese populations involving 1,785,569 participants showed an anti-HEV IgG seroprevalence of 23.17% [11]. Another analysis of HEV serology data from Chongqing, China, for 10 consecutive years from 2012 to 2021 showed an increase in Hepatitis E IgG antibody positivity from 1.61% to 50.63% [12]. In addition, seropositivity surveys of HEV have also been conducted in pregnant women [13] and blood donors [14] that showed varying degrees of risk of infection and transmission.

Domestic pigs and wild boars are the main hosts of HEV-4, and the anti-HEV IgG seropositivity rate of swine herds ranges from 8 to 93% [15]. Blood samples from pig farms in Guangdong, China, collected from 2014 to 2016 showed that the average positivity rate for HEV antibodies was 69.2% [4]. Pigs may be infected with HEV at various stages of growth but most commonly between 8 and 15 weeks of age [16,17,18]. The seroconversion of Hepatitis E antibodies in pigs usually occurs between the 13th and 17th week of age, followed by a weakening of maternal antibodies, with anti-HEV IgM and IgA peaking first, followed by anti-HEV IgG.

The consumption of contaminated pork products is an important route of HEV infection [19,20]. Therefore, understanding the presence and dynamics of the virus in swine is essential for developing infection prevention measures to protect occupational exposure and public health. In 2000–2002, an anti-HEV antibody test conducted on pigs in Guangdong showed a seropositivity rate of 89.1% [21], and a study conducted in 2008 showed an HEV antibody positivity rate of 98% [22]; however, there have been limited further reports on the prevalence of serum HEV antibodies. Therefore, this study was conducted to provide further data on the distribution and prevalence of anti-HEV IgG in pigs in Guangdong, with the ultimate aim of revealing the risk of infection and providing a reference for the prevention and control of foodborne zoonotic pathogens.

## 2. Materials and Methods

### 2.1. Sample Collection and Processing

**Figure 1:**
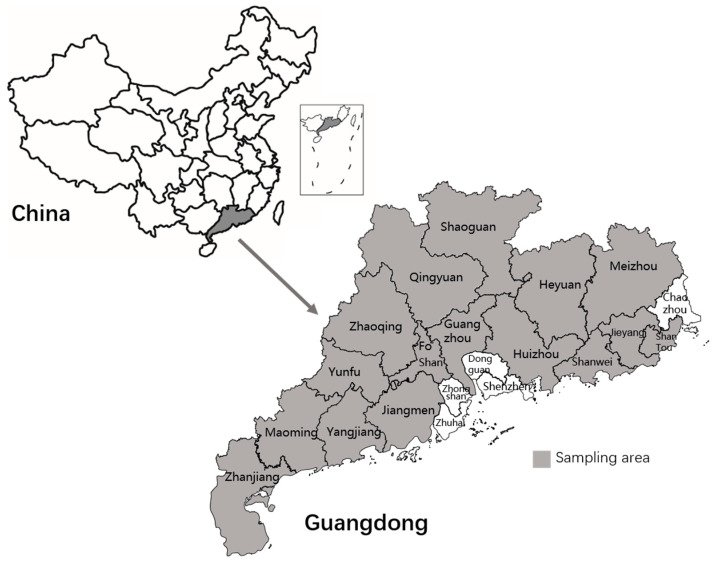
Map showing the Guangdong Province in China and expanded map of the province showing the sampling area in gray.

A total of 1568 pig blood samples were collected from 25 pig farms in 16 cities across the Guangdong Province (Jieyang, Meizhou, Shanwei, Shantou, Yunfu, Yangjiang, Maoming, Zhanjiang, Shaoguan, Heyuan, Qingyuan, Guangzhou, Foshan, Jiangmen, Zhaoqing, and Huizhou) (Figure 1). The samples were collected over 3 years: in 2022, 337 samples were collected from five industrial pig farms; in 2023, 1107 samples were collected from twenty industrial pig farms; and in 2024, 84 samples were collected from only one industrial pig farm in Heyuan city. The farm was sampled in April and September 2023 and in January 2024, which assisted in understanding the dynamics of HEV infection on the farm. The total sample size was calculated based on the prevalence of previous tests and distributed to pig farms in each region of Guangdong according to the principle of probability proportionate to size sampling. Specific information on the type, size, and number of samples collected from pig farms is provided in Supplementary Table S1.

The samples were categorized and labeled accordingly. Following 10 min centrifugation at 3000 rpm, the sera were collected and stored at −20 °C for further testing. To study the dynamics of the viral response at various stages of development, 1070 pig samples with detailed records about age were chosen for further research. 

### 2.2. HEV IgG Antibody Detection

Anti-HEV IgG was detected in accordance with the manufacturer’s instructions using the HEV IgG Antibody Diagnostic Kit (Beijing Wantai Biopharmaceutical Co., Ltd., Beijing, China), an enzyme-linked immunosorbent assay (ELISA) with a specificity and sensitivity of 97.96% and 99.9%, respectively. The kit was produced using a recombinant protein that corresponds to amino acids’ 394–606 region of the major structural protein covered by the open reading frame 2 of the HEV genome.

### 2.3. Subgroup Analysis

To investigate the effects of HEV on different populations, all samples were classified by year (2022, 2023, and 2024), season (spring: March to May, summer: June to August, autumn: September to November, and winter: December to February), region (western Guangdong: Zhanjiang, Maoming, Yangjiang, and Yunfu; eastern Guangdong: Jieyang, Meizhou, Shantou, and Shanwei; northern Guangdong: Shaoguan, Qingyuan, and Heyuan; and Pearl River Delta: Guangzhou, Foshan, Jiangmen, Zhaoqing, and Huizhou), and age (piglet: 0–4 weeks, weaner: 5–8 weeks, grower: 9–22 weeks, gilt: unproduced, sow: produced, and boar: male breeding stock) for subgroup analysis.

### 2.4. Data–Statistical Analysis

The data and statistical analyses of HEV prevalence were estimated from the ratio of positive samples to the total number of samples analyzed, with a binomial confidence interval of 95%. Frequencies were compared using the chi-squared test, and exposure risk factors were analyzed using a multifactor binomial logistic regression analysis, with a *p*-value < 0.05 being considered significant. The data were analyzed using Microsoft Excel 2019 and SPSS version 27.0.

### 2.5. Ethics Statement

All procedures involving the samples collected in this study were conducted in strict accordance with the guidelines of the Animal Ethics Committee of the Institute of Animal Health, Guangdong Academy of Agricultural Sciences.

## 3. Results

### 3.1. HEV Seroprevalence in Domestic Pigs

In domestic pigs, the total anti-HEV IgG rate was 57.53% (902/1568, 95% CI = 55.0–60.0). There were no positive samples in only one farm herd in Shaoguan (0/10; 95% CI = 0–30.8). Pigs from 24 of the 25 farms tested positive for HEV, and the prevalence of anti-HEV IgG in the herd was 96% (24/25, 95% CI = 79.6–99.9%). The seropositivity rates varied according to the location tested (Table 1), ranging from 11.67 to 100%. Notably, the two cities showing 100% seropositivity may be related to insufficient sampling. 

### 3.2. Analysis of Risk Factors of HEV Seroprevalence in Different Subgroups

Data statistics were obtained to analyze the risk factors for subgroups with different positivity rates. The year, season, region, and age were all linked risk factors for HEV in Guangdong, with the season and region showing more significant impacts according to binary logistic regression analysis.

#### 3.2.1. Subgroup by Year

**Figure 2:**
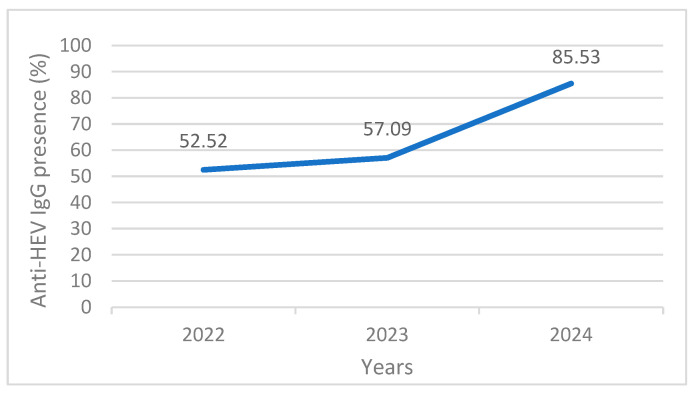
Anti-HEV IgG positive test results by year.

The pig HEV seroprevalence ranged from 52.52 to 85.53% between 2022 and 2024. The 2024 samples showed the highest positive rate, with 85.53% (72/84, 95% CI = 76.4–92.4) of seroprevalence. In 2022 and 2023, the HEV seropositivity rates were 52.52% (198/377, 95% CI = 47.3–57.7), and 57.09% (632/1107, 95% CI = 54.1–60.0), respectively (Figure 2). Compared with 2022 (Table 2), the risk of infection was 5.424 times higher in 2024 (OR = 5.424, 95% CI = 2.850–10.325, *p* < 0.001). 

#### 3.2.2. Subgroup by Season

The seroprevalence of pig HEV varied across the seasons, ranging from 33.93 to 75.91% (Table 3). The seroprevalence was lowest in spring, with a seropositivity rate of 33.93% (208/613, 95% CI = 30.2–37.8), while the highest was observed in autumn, with a seropositivity rate of 75.91% (293/386, 95% CI = 71.3–80.1). Compared with spring, the risk of infection was 3.708 times higher in summer (95% CI = 2.746–5.007, *p* < 0.001), 6.134 times higher in autumn (95% CI = 4.604–8.174, *p* < 0.001), and 5.841 times higher in winter (95% CI = 4.277–7.978, *p* < 0.001). This indicated that the risk of infection was lowest in spring and significantly greater in summer, autumn, and winter, with autumn and winter showing the most significant differences.

**Figure 3:**
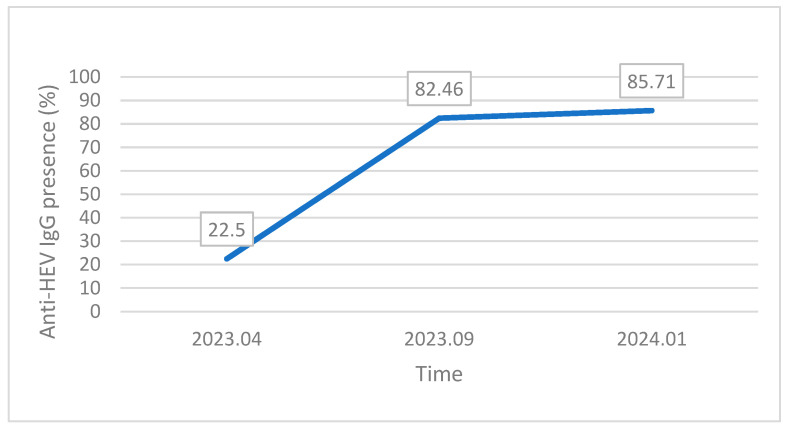
Results of three samples taken at one pig farm in Heyuan city.

Three samples were taken at one pig farm in Heyuan city: spring (April 2023), autumn (September 2023), and winter (January 2024). The seropositivity rates of these samples were 22.5% (18/80, 95% CI = 13.9–33.2), 82.46% (47/57, 95% CI = 70.1–91.3), and 85.71% (72/84, 95% CI = 76.4–92.4), respectively (Figure 3). This was consistent with our finding that the risk of infection was lowest in spring and highest in autumn and winter.

#### 3.2.3. Subgroup by Region

The seroprevalence of porcine HEV varied across different regions, ranging from 40.96 to 77.82% (Table 4). The lowest positivity was seen in western Guangdong at 40.96% (213/520, 95% CI = 36.7–45.3) and the highest in eastern Guangdong at 77.82% (193/248, 95% CI = 72.1–82.8). Compared to western Guangdong, the infection risk was 5.058 times higher in eastern Guangdong (95% CI = 3.575–7.155, *p* < 0.001), 2.008 times higher in northern Guangdong (95% CI = 1.545–2.609, *p* < 0.001), and 2.806 times higher in the Pearl River Delta region (95% CI = 2.134–3.689, *p* < 0.001). These data suggest regional variations in HEV infection, with the lowest risk occurring in western Guangdong. Eastern Guangdong, northern Guangdong, and Pearl River Delta were all significantly higher in terms of infection risk than western Guangdong.

#### 3.2.4. Subgroup by Age

The seroprevalence of pig HEV varied by age group and ranged from 41.01% to 62.41% (Table 5). Grower pigs demonstrated the lowest prevalence at 41.01% (73/178, 95% CI = 33.7–48.6), while sows had the highest prevalence at 62.41% (73/178, 95% CI = 33.7–48.6). The infection risk was 1.602 times higher in gilts (95% CI = 1.046–2.453, *p* < 0.001) than in growers, and 2.388 times higher in sows (95% CI = 1.672–3.412, *p* < 0.001). Piglets, weaners, and boars did not exhibit any statistically significant differences. These results suggest that age influences HEV infection, with a heightened risk in sows and gilts.

## 4. Discussion

Numerous studies have confirmed that HEV is widespread in pig herds, including on large-scale pig farms, in slaughterhouses, and in wild boars [20,23]. HEV has also been detected in retail pork and pork liver products [24,25,26], indicating the potential for zoonotic transmission. However, the relatively high rate of HEV positivity in China may pose a greater risk to pork production and food safety.

To study the dynamics of HEV infection, we used data spanning three years in the Guangdong Province and involving 25 different farms across 16 cities. The results showed an overall seropositivity rate of 57.53% (902/1568, 95% CI 55.0–60.0) among domestic pigs, which complements observations from recent studies conducted in the Guangdong Province. In Guangdong, Zhang et al. discovered an average HEV positivity of 71.9% among pig farms in various regions from 2009 to 2011 [27]. Subsequently, Liang et al. surveyed anti-HEV antibodies in pigs between 2011 and 2013 and reported a prevalence of 64.7% [2]. Compared to prior studies across Guangdong’s pig farms, our report shows diminished seropositivity rates, and this could possibly be attributed to effective farm management strategies and stringent disease prevention protocols combined with heightened staff vigilance. In contrast, compared to 2022 and 2023, the seroprevalence observed in 2024 was significantly higher in our study, suggesting an upsurge in HEV infection among pigs. However, it is possible that these results may not be representative because the sample size in 2024 was relatively small, and sampling was conducted on only one farm in a single region where seropositivity was also very high in 2023.

Our findings were similar to those of a survey conducted by Duan et al. in the Chuxiong area of Yunnan. In their study, the overall seropositivity rate for anti-HEV IgG in pig sera was 56.35%, with a seropositivity rate of 51.68% on breeding farms [28]. Compared with previous studies in Tibet [29] and Hubei [30], a higher seropositivity rate was found in our study. This difference could be attributed to variations in the detection methods used and the varying levels of infection in different regions. The seropositivity rates for anti-HEV IgG in pig farms in 16 different cities in Guangdong ranged from 11.67% to 100% (Table 1). Differences in seropositivity rates suggest variations in transmission dynamics or routes of exposure to HEV among the pig populations studied in different regions. Similarly, Zhang et al. [31] found variations in infection rates among different regions of 21 cities and prefectures in the Sichuan Province from 2019 to 2020. 

We found that HEV seroprevalence in pigs varied between seasons, ranging from 33.93% to 75.91%. The lowest seropositivity rate was observed in spring samples and the highest in autumn samples. This indicates that the risk of HEV infection varies seasonally, with a lower risk in spring and a higher risk in summer, autumn, and winter. A risk factor analysis showed that the season was a risk factor for HEV infection, differing from Zheng’s findings [32] of a lower risk of infection during autumn. HEV is transmitted via the fecal–oral route and is detectable at 4 °C for as long as 70 days [33]. A cell culture system infected with a heated fecal suspension of HEV showed that the virus was inactivated at 56 °C in only 15 min [34], so it has been hypothesized that the survival and infectivity of HEV in the environment may be related to seasonal temperature changes. However, the successful infection of pigs with HEV-positive swine liver homogenates showed that incubation at 56 °C for 1 h was not effective in inactivating HEV [35], so it has also been hypothesized that the seasonal pattern of HEV prevalence may be attributed to factors other than temperature, such as the medium in which it is present (water, food, etc.) [36]. We hypothesize that this difference between seasons may be related not only to temperature but also to precipitation. Guangdong belongs to the East Asian monsoon zone, where rain and heat occur together. Spring is drier and cooler than summer and autumn. This may have contributed to the seasonal differences in our findings. Additionally, rainfall in Guangdong is mainly concentrated between April and September. Yangjiang city is a precipitation center, and it exhibited high seroprevalence rates (75%, 15/20), suggesting a possible association with precipitation. However, further research is needed to determine the close relationship between temperature, precipitation, and HEV infection rate.

Our study found that the seroprevalence of HEV in pigs varied across different regions, ranging from 40.96 to 77.82%. The lowest and highest seropositivity rates were observed in samples collected from western Guangdong and eastern Guangdong, respectively. Therefore, the risk of infection was lowest in the western region, whereas the eastern, northern, and Pearl River Delta regions had significantly higher seroprevalence rates, with the eastern region showing the most significant difference. These differences may be related to factors such as terrain and water flow. Overall, the elevation in Guangdong is higher in the north and west and lower in the east and south. This difference in elevation may change viral infectivity in the environment. Furthermore, many rivers in Guangdong originate in mountainous areas; the western and northern regions are located upstream, whereas the eastern and Pearl River Delta regions are located downstream, which may also contribute to the different seroprevalence rates observed in pig populations. Similarly, Liang et al. found that the Guangdong swine HEV epidemic might have spread primarily through the Pearl River water system. However, further research is needed to determine the close relationship between terrain, water flow, and HEV infection rate.

Lu et al. [37] found that various factors, such as climate, environment, population mobility, and lifestyle, can influence the spread of viruses. Typically, enteric infectious diseases have a high incidence rate during summer (May to October). Yu et al. [38] showed a correlation between climatic factors and the prevalence of enteroviruses, with specific climatic factors acting as drivers of viral spread. The link with food is more significant for viruses transmitted via the fecal–oral route. Shanshan et al. [39] reported a small-scale acute Hepatitis E outbreak in a recruiting company in Guangzhou, Guangdong, and found that the outbreak may be due to close contact and shared meals. Miao et al. [40] also investigated an outbreak of Hepatitis E in 2022 in a nursing home in the Zhejiang Province. The consumption of undercooked pig liver may have caused this outbreak due to poor cooking practices and hygiene. This finding highlights the importance of focusing on the relationship between food consumption and viral transmission. Therefore, it is essential to consider multiple factors, including the climate, environment, population dynamics, and cultural practices, when studying the spread and transmission patterns of viruses.

Among the age groups, sows (269/431, 62.4%) were found to have the highest seroprevalence of HEV IgG, whereas the seroprevalence of HEV IgG was the lowest in growing pigs (9–22 weeks) (73/178, 41.0%). This agrees with previous observations that found pigs are infected between the growing stage (8–15 weeks) and that anti-HEV IgG appears later after infection [18]. These results are also similar to those of Liang et al., who reported a higher risk of HEV infection in growing pigs, sows, and boars than in nursery pigs [2]. A study by Pavia et al. [41] that compared pigs of all ages found the highest seropositivity rate (64.5%) in pigs > 4.5 months. Tsachev et al. [42] reported the highest HEV positivity rate (80%) in sows. This is similar to our findings of the highest positivity rates in gilts and sows, although their rates were higher, possibly due to regional and temporal variations in sampling. In addition, several factors can significantly delay HEV seroconversion, such as concurrent infection with the Porcine Reproductive and Respiratory Syndrome Virus and the presence of maternal antibodies. These factors may result in the presence of HEV RNA at slaughter, posing a risk of human infection through the consumption of contaminated food [43].

Numerous studies have shown a correlation between human and swine HEV, indicating the zoonotic nature of the disease [44,45]. A high frequency of anti-HEV antibodies has been found in veterinary and pig farm workers, suggesting that occupational exposure is one of the risks of HEV infection. Another route of HEV transmission to the general population is the consumption of undercooked pork and game meat [25,46]. In this respect, studies have demonstrated the widespread prevalence of HEV infection in humans. Liang et al. conducted an epidemiological study from 2011 to 2013 on pig farmers and healthy individuals in Guangdong and found that 48.25% of the pig farmers and 38.34% of the general population tested positive for anti-HEV IgG. Seroprevalence rates have also been stratified according to age [2]. Li et al. [47] analyzed 19,933 blood samples collected in Guangdong in 2018 for HEV-IgG and found a seropositivity rate of 27.82%. They observed variations in the seroprevalence rates among different sexes and age groups, and they noted regional variations, where Yangjiang city had the highest rate (52.48%). This study found a seropositivity rate of 75% (15/20) in the pig population in Yangjiang city, indicating a high level of infection. Therefore, it is important to investigate whether a high seropositivity rate in humans is also associated with high seropositivity rates in pigs. Lu [48] observed a peak incidence of HEV in humans from January to April and estimated that the exposure period occurred around the Chinese New Year. The increased risk during this period can be attributed to factors such as increased social gatherings leading to foodborne transmission. Moreover, seasonal variations in the detection rate of HEV RNA in pig bile were observed, with the highest rates occurring in September and October and the lowest rates in May and June. The peak and trough occurred approximately three months before human HEV cases, suggesting the possibility of transmission of HEV from pig populations to humans. This finding warrants future research. 

In conclusion, we observed variations in the seroprevalence of HEV in pigs from different regions of Guangdong, China. Year, season, region and age were all relevant risk factors, and seasonal and regional variations may be attributed to environmental factors. Understanding seasonal and geographic patterns of HEV distribution has important public health implications for preventing disease transmission from pigs to humans. Further epidemiologic investigations among humans in the same geographic region are therefore needed to assess potentially similar seasonal patterns of human HEV infection. In addition, since pork is the main meat consumer product for the Chinese people, special attention should also be paid to the hygiene of pork production and cooking methods to minimize foodborne infections of HEV.

## 5. Conclusions

HEV is a zoonotic pathogen that is prevalent worldwide. Although animal infections are mostly asymptomatic, they can cause acute and chronic viral hepatitis in humans. This study evaluated the prevalence of anti-HEV IgG in farms in the Guangdong Province, and the overall positivity rate was more than half of the farms. The seroprevalence in the pig herds was 96%. Owing to the high seropositivity rate, it is likely that almost all of the participating farms in Guangdong’s pig herds are exposed to HEV. Future research should examine factors associated with the risk of HEV on farms, conduct further epidemiologic investigations in humans in the same geographic area, and understand seasonal and geographic patterns of HEV distribution to prevent the introduction and spread of the virus on farms. This is of significant public health importance to protect the safety of the relevant occupational groups and to prevent the spread of zoonotic pathogens from farms.

## Figures and Tables

**Table 1:** Anti-HEV IgG among pigs from different cities in Guangzhou showing a 95% CI confidence interval.

City	No. Farms	No. Positive Farm	No. Samples	No. Positive Samples	Sample Prevalence (95% CI)
Jieyang	2	2	103	63	61.17% (51.1–70.6)
Meizhou	1	1	71	58	81.69% (70.7–89.9)
Shanwei	1	1	70	68	97.14% (90.1–99.7)
Shantou	1	1	4	4	100% (39.8–100)
Yunfu	2	2	393	124	31.55% (27.0–36.4)
Yangjiang	2	2	20	15	75% (50.9–91.3)
Maoming	1	1	12	12	100% (73.5–100)
Zhanjiang	2	2	95	62	65.26% (54.8–74.7)
Shaoguan	2	1	95	24	25.26% (16.9–35.2)
Heyuan	2	2	261	166	63.60% (57.4–69.4)
Qingyuan	2	2	58	51	87.93% (76.7–95.0)
Guangzhou	1	1	90	51	56.67% (45.8–67.1)
Foshan	2	2	57	53	92.98% (83.0–98.1)
Jiangmen	1	1	60	7	11.67% (4.8–22.6)
Zhaoqing	1	1	87	65	74.71% (64.3–83.4)
Huizhou	2	2	92	79	85.87% (77.0–92.3)
Total	25	24	1568	902	57.53% (55.0–60.0)

**Table 2:** Time factor affecting HEV seroprevalence with a 95% CI confidence interval; OR is the odds ratio.

Year	No. Tested	No. Positive	Prevalence (95% CI)	OR (95% CI)	*p*-Value
2022	377	198	52.52% (47.3–57.7)	Reference	
2023	1107	632	57.09% (54.1–60.0)	1.203 (0.951–1.521)	0.123
2024	84	72	85.71% (76.4–92.4)	5.424 (2.850–10.325)	<0.001

**Table 3:** Seasonal factors affecting HEV seroprevalence with a 95% CI confidence interval; OR is the odds ratio.

Season	No. Tested	No. Positive	Prevalence (95% CI)	OR (95% CI)	*p*-Value
Spring	613	208	33.93% (30.2–37.8)	Reference	
Summer	273	179	65.57% (59.6–71.2)	3.708 (2.746–5.007)	<0.001
Autumn	386	293	75.91% (71.3–80.1)	6.134 (4.604–8.174)	<0.001
Winter	296	222	75.00% (69.7–79.8)	5.841 (4.277–7.978)	<0.001

**Table 4:** Regional factors affecting HEV seroprevalence with a 95% CI confidence interval; OR is the odds ratio.

Region	No. Tested	No. Positive	Prevalence (95% CI)	OR (95% CI)	*p*-Value
Western Guangdong	520	213	40.96% (36.7–45.3)	Reference	
Eastern Guangdong	248	193	77.82% (72.1–82.8)	5.058 (3.575–7.155)	<0.001
Northern Guangdong	414	241	58.21% (53.3–63.0)	2.008 (1.545–2.609)	<0.001
Pearl River Delta	386	255	66.06% (61.1–70.8)	2.806 (2.134–3.689)	<0.001

**Table 5:** Age factor affecting HEV seroprevalence, with a 95% CI confidence interval; OR is the odds ratio.

Age	No. Positive	No. Tested	Prevalence (95% CI)	OR (95% CI)	*p*-Value
Piglet	67	152	44.08% (36.0–52.4)	1.134 (0.732–1.757)	0.574
Weaner	53	112	47.32% (37.8–57.0)	1.292 (0.803–2.080)	0.291
Grower	73	178	41.01% (33.7–48.6)	Reference	
Gilt	88	167	52.69% (44.8–60.5)	1.602 (1.046–2.453)	0.030
Sow	269	431	62.41% (57.7–67.0)	2.388 (1.672–3.412)	<0.001
Boar	16	30	53.33% (34.3–71.7)	1.644 (0.756–3.575)	0.207
Totals	566	1070	52.90% (49.9–55.9)		

## Data Availability

Primary data used in this paper are available from the authors upon request (cairujian@gdaas.cn).

## Supplementary Materials

The following supporting information can be downloaded at: https://www.mdpi.com/article/10.3390/ani14131861/s1, Table S1. Anti-HEV IgG among pigs from different farms in Guangzhou.
