# Supplementary material for: Seroprevalence survey of Hepatitis E Virus in Domestic Pigs in Guangdong, China
Source: Animals (Basel). 2024 Jun 24;14(13):1861. doi: 10.3390/ani14131861 (PMC11240697; doi:10.3390/ani14131861)
Supplement: Supplementary file 1 [file animals-14-01861-s001.zip › animals-3032983-supplementary.pdf]

Supplementary Table S1. Anti-HEV IgG among pigs from different farms in Guangzhou.

Farm	Farm Region	Type of Farm	Size of Farm	No. Samples	No. Positive Samples	Samples Prevalence (95%CI)
Farm1	Western Guangdong	Farrow-to-Finish	>5000	293	78	26.62% (21.6-32.1)
Farm2	Western Guangdong	Farrow-to-Finish	2000-5000	55	37	67.27% (53.3-79.3)
Farm3	Western Guangdong	Finisher	< 2000	10	9	90.00% (55.5-99.7)
Farm4	Western Guangdong	Finisher	< 2000	10	6	60.00% (26.2-87.8)
Farm5	Western Guangdong	Farrow-to-Finish	>5000	100	46	46.00% (36.0-56.3)
Farm6	Western Guangdong	Finisher	< 2000	40	25	62.50% (45.8-77.3)
Farm7	Western Guangdong	Finisher	< 2000	12	12	100.00% (73.5-100.0)
Farm8	Eastern Guangdong	Farrow-to-Finish	< 2000	50	15	30.00% (17.9-44.6)
Farm9	Eastern Guangdong	Farrow-to-Finish	>5000	53	48	90.57% (79.3-96.9)
Farm10	Eastern Guangdong	Farrow-to-Finish	2000-5000	71	58	81.69% (70.7-89.9)
Farm11	Eastern Guangdong	Farrow-to-Finish	2000-5000	70	68	97.14% (90.1-99.7)
Farm12	Eastern Guangdong	Finisher	< 2000	4	4	100.00% (39.8-100.0)
Farm13	Northern Guangdong	Farrow-to-Finish	>5000	221	137	61.99% (55.2-68.4)
Farm14	Northern Guangdong	Farrow-to-Finish	>5000	85	24	28.24% (19.0-39.0)
Farm15	Northern Guangdong	Farrow-to-Finish	2000-5000	40	29	72.50% (56.1-85.4)
Farm16	Northern Guangdong	Finisher	< 2000	10	0	0% (0.0-30.8)
Farm17	Northern Guangdong	Farrow-to-Finish	< 2000	26	25	96.15% (80.4-99.9)
Farm18	Northern Guangdong	Farrow-to-Finish	2000-5000	32	26	81.25% (63.6-92.8)
Farm19	Pearl River Delta	Finisher	< 2000	5	3	60.00% (14.7-94.7)

Farm20	Pearl River Delta	Farrow-to-Finish	>5000	60	7	11.67%（4.8-22.6）
Farm21	Pearl River Delta	Farrow-to-Finish	>5000	87	65	74.71%（64.3-83.4）
Farm22	Pearl River Delta	Finisher	< 2000	90	51	56.67%（45.8-67.1）
Farm23	Pearl River Delta	Farrow-to-Finish	2000-5000	52	50	96.15%（86.8-99.5）
Farm24	Pearl River Delta	Farrow-to-Finish	>5000	52	51	98.08%（89.7-100.0）
Farm25	Pearl River Delta	Farrow-to-Finish	2000-5000	40	28	70.00%（53.5-83.4）
